# Socioeconomic, sex and area related inequalities in childhood stunting in Mauritania: Evidence from the Mauritania Multiple Indicator Cluster Surveys (2007–2015)

**DOI:** 10.1371/journal.pone.0258461

**Published:** 2021-10-18

**Authors:** Gebretsadik Shibre, Betregiorgis Zegeye, Gorems Lemma, Birhan Abebe, Gashaw Garedew Woldeamanuel

**Affiliations:** 1 Department of Reproductive, Family and Population Health, School of Public Health, Addis Ababa University, Addis Ababa, Ethiopia; 2 HaSET Maternal and Child Health Research Program, Shewarobit Field Office, Shewarobit, Ethiopia; 3 Chacha Health Center, Angolela Tera Health Office, Debre Birhan, Ethiopia; 4 Department of Environmental and Health Safety, Habesha Brewery, Debre Birhan, Ethiopia; 5 Department of Biomedical Sciences, School of Medicine, College of Medicine and Health Sciences, Wolkite University, Wolkite, Ethiopia; University of Washington, UNITED STATES

## Abstract

**Introduction:**

The prevalence of stunting in under five children is high in Mauritania. However, there is a paucity of evidence on the extent and the overtime alteration of inequality in stunting. To this end, we did this study to investigate stunting inequality and the change with time using three rounds of Mauritania Multiple Indicator Cluster Surveys. The evidence is important to inform implementation of equitable nutrition interventions to help narrow inequality in stunting between population groups.

**Methods:**

World Health Organization’s (WHO) Health Equity Assessment Toolkit (HEAT) was used in the analysis of stunting inequality. Following standard equity analysis methods recommended by the WHO, we performed disaggregated analysis of stunting across five equity stratfiers: Wealth, education, residence, sex and sub-national regions. Then, we summarized stunting inequality through four measures of inequality: Difference, Ratio, Population Attributable Fraction and Population Attributable Risk. The point estimates of stunting were accompanied by 95% confidence intervals to measure the statistical significance of the findings.

**Results:**

The national average of childhood stunting in 2007, 2011 and 2015 was 31.3%, 29.7% and 28.2%, respectively. Glaring inequalities in stunting around the five equity stratifiers were observed in all the studied periods. In the most recent survey included in our study (2015), for instance, we recorded substantial wealth (PAF = -33.60; 95% CI: -39.79, -27.42) and education (PAF = -5.60; 95% CI: -9.68, -1.52) related stunting inequalities. Overall, no substantial improvement was documented in wealth and sex related inequality in stunting between 2007 and 2011 while region-based inequality worsened during the same time periods.

**Conclusions:**

The burden of stunting appeared to be heavily concentrated among children born to socioeconomically worse-off women, women who live in rural settings and certain subnational regions. Targeted nutrition interventions are required to address drivers of stunting embedded within geographic and socioeconomic contexts.

## Introduction

Stunting is one of the most common manifestations of undernutrition and refers to being too short in length or height for certain age [1]. Stunting inside the first 1000 days is particularly devastating as its impacts are carried into later in life. Stunted children can suffer permanent physical and cognitive impairment, and poor performance at school, low adult salaries, loss of productivity, and chronic diseases during adulthood period [1, 2]. What is even worse is that, the damaging impact of stunting can continue in population for a long time and even persist into the next generation [1]. Multiple factors such as inadequate nutrition, recurrent infection, and insufficient psychosocial stimulation interact together to lead to impaired child linear growth [2].

Globally, stunting affected 144 million under five children in 2019, translated to 21.3% of the world’s children age under five [1]. Breaking down the global average estimates reveals that Asia and Africa remain the hardest hit regions, with respectively 54% and 40% of stunted children reported from the two continents [1].The Eastern Africa, Middle Africa and Southern Asia are particularly the highest stunting burden sub-regions globally, where more than 30% of the under five children in each of these sub-regions are stunted [1]. Despite stunting declined globally between 2000 and 2019, the pace of reduction of stunting has seen huge disparity worldwide. Astonishingly, Africa is the only region that has seen increased number of stunted populations during the past nearly two decades [1].

Mauritania is a country with a high burden of malnutrition among its under-five children. In 2015, the national prevalence of stunting among children age less than five years was 27.9%, which is slightly higher than the stunting average of 25% for developing nations [3]. Currently, Mauritania is off course to hit the global stunting target [3], indicating that more work is needed to meet the target. Not only is Mauritania a high stunting burden country, but there is substantial within country inequality in stunting according to the place of residence, sex, subnational regions, and socioeconomic status [3, 4].

Our literature review showed that few studies have attempted to shed light on the burden of stunting in Mauritania and the variation by different population subgroups. To this end, this paper aimed to assess stunting inequality and the overtime alteration across the five dimensions of inequality following internationally approved methods for equity study. The findings from this inequality analysis can largely contribute in facilitation of reduction of stunting disparities [5].

## Materials and methods

### Brief overview of the study setting

Mauritania is a country with huge pastoral land and only less than one percent of land is capable of being ploughed and used to grow crops [6]. As of the 2018, Mauritania has a population of about 4 million with population density of 3.9 inhabitants per square kilometer. Mauritania’s economy has been on rise between 2015 and 2018, driven mainly by the healthy activities in the sector of telecommunications, transport, electricity, and primary sectors [6]. The sustained economic growth in the country has resulted in the reduction of the proportion of poor, which fell by 11.5 percentage points between 2008 and 2014. Similarly, according to the measure of Gini coefficient, income inequality has decreased over the last few years. In terms of other social indicators, however, the country is still lagging behind. For instance, only slightly higher than a half of children aged 6 to 11 attend primary school, 33% of households live in unwarranted housing, and only 38% of the population has access to electricity [6].

Childhood mortality is high in Mauritania. In 2018, nearly 76 children age under five out of 1000 live births die before celebrating their fifth birth day, which is higher as compared to the world average (28 deaths per 1000 live births) with the death rate being slightly higher among male children. Infant and neonatal mortality rates are respectively 52 and 33 deaths per 1000 live births ([]. The high mortality rates among children can be associated with low utilization of basic maternal health care services. The proportion of mothers who receive four or more antenatal care visits during pregnancy is only 63%, and mothers who get postnatal care are even lower, 57%. Further, about one in three mothers still gives birth at home [7].

### Data source

The source of our analysis is the offline version of the WHO HEAT software. The detail discussion of the software has been available elsewhere [8, 9]. But in brief, the HEAT is software that enables examination and analysis of health inequalities within and between countries. The software is tremendously valuable to explore the health disparity situation in a more systematic detail. The HEAT software application comprises of the WHO Health Equity Monitor (HEM) database [10]. The database stores data coming from Demographic and health Survey (DHS) and Multiple Indicator Cluster Survey (MICS) conducted in many low-or-middle income countries including Mauritania. Currently, the database provides detail inequality assessment for more than 30 Reproductive, Maternal, Newborn and Child health indicators.

For the present study, we used the dataset derived from the three waves of the Mauritania Multiple Indicator Cluster Surveys (MMICSs) conducted in 2007, 2011 and 2015 that are found in the software. The MMICS is a nationally representative survey designed to collect information on various health topics such as nutrition, unmet needs, female genital mutilation, domestic violence, access to the mass media, fertility, young child development, breastfeeding and food intake, vaccinations, and treatment of diseases. By providing the government of Mauritania with valid and up-to-date health indicators on women age 15–49, men age 15 to 49 and children under 5, the survey aims to monitor and assess the health situation of the population. The sample design of the survey is meant to provide estimates on several health indicators at national level, as well as at urban and rural areas and for 13 Wilayas. The detailed sampling methodology of the surveys has been described in detail in the respective survey reports [11–13].

### Variables and their measurements

Stunting is the primary variable of interest for the study. Stunting was measured as height-for-age (HAZ) less than minus 2 Standard Deviation (-2SD) from the median of the WHO child growth standard [14]. For calculation of the percentage of under five children that are stunted, the HAZ scores were recoded so that children whose HAZ falls between less than –2 SD and -6 SD from the WHO reference population are coded 1 and HAZ that lies between -2 SD and + 6 SD are coded as 0. The analysis was carried out on children who born five years preceding the survey.

Inequality in stunting was measured for five equity stratifiers. Child sex (female versus male), maternal educational level was classified as no formal education, primary school, secondary school and above, economic status was approximated through a wealth index. Wealth index is customarily computed using a durable goods, household characteristics and basic services following the methodology explained elsewhere [15]. Though the type of asset variables used for constructing wealth index vary between surveys [16], the commonly used variables include water and sanitation facilities (WASH), radio, television, types of materials used to make floor, roof and wall of a household, car, bicycle, motorcycle, and electricity [15]. It has also been shown that, any indicator or variable that is deemed important for indicating economic status of households can be used in the construction of wealth index [15]. The constructed wealth index is then divided into five quintiles: poorest (quintile 1), quintile 2, quintile 3, quintile 4 and richest (quintile 5). Place of residence as urban versus rural. The sub-national region included the 13 regions in the country.

### Data analysis

As we briefly described in the data source sub-section above, the offline version of the WHO HEAT software updated in 2019 was used for analysis [17]. Analysis was done using two main steps. First, prevalence of stunting was disaggregated by the above mentioned five equity stratifiers, i.e., child sex, maternal educational level, household wealth index, place of residence, and subnational region. Second, stunting inequality was further analyzed using the four summary measures of health inequality: Difference (D), Ratio (R), Population Attributable Risk (PAR) and Population Attributable Fraction (PAF). The choice of the summary measures for an inequality study should be based on the fact that, the selected summary measures need to be of simple and complex measures [5]. At the same time, summary measures need to be relative and absolute measures to be able to examine inequality from different angles. For our study, we chose measures of inequality in accordance with this recommendation. While the “D” and “R” are simple measures, the PAR and PAF are complex measures [5]. Moreover, the “D” and “PAR” are absolute measures, and the R and PAF are relative measures. The simple measures of health inequality are used to compare health indicators between two groups, and are useful choices for dimensions of inequality such as place of residence and sex. For dimensions of inequality with more than two categories such as wealth and education, however, more complex measures are required that account for the entire subpopulations in all the categories though simple measures can still be used.

The detail elucidation about the summary measures adopted in the present study has been clearly made elsewhere [5, 17]. Briefly, for education and economic status, D was calculated as follows.


$$D=y_{high}‐y_{low}$$

Specifically for wealth quintile and maternal educational level, D was calculated stunting in the poorest group minus the richest group, and stunting in the non-educated group minus the secondary education and above group. For place of residence, it was calculated as stunting in rural minus in the urban settings. For the sub-national regions, D was calculated as stunting in the region with the highest stunting burden minus the region with the lowest stunting. Ratio (R) was calculated as the ratio of two subgroups:

$$R=Y_{high}/Y_{low}.$$

For place of residence, Y_high_ and Y_low_ refers to rural and urban settings, respectively. In educational status, Y_high_ and Y_low_ refers to the least advantaged subgroups (no education) and the most advantaged subgroups (secondary schools or higher), respectively. For economic status, Y_high_ and Y_low_ refer to the poorest quintile and the richest quintile, respectively. For sex, the calculation was performed as the ratio of stunting in male children (Y_high_) to female children stunting (Y_low_). For the sub-national regions, R was calculated as the region with the highest stunting burden divided by the region with the lowest stunting prevalence. When there is no inequality between any two groups, D assumes a value of 0 and R becomes one.

PAR was calculated as the difference between the stunting estimate for the reference subgroup (Yref) and the national average of childhood stunting (μ):

PAR=Yref‐μ.

The Yref varies depending on the type of dimensions of in equality. In our study, Yref refers urban setting for place of residence, secondary education for education and richest sub-group for economic dimensions. For the sub-national region regions, Yref refers to the sub-national region with the lowest estimate of stunting. Once PAR is calculated this way, PAF is calculated as PAR/ μ and multiplied by 100.


PAF=[PAR/μ]*100

The PAR and PAF measures become zero when there is no inequality between the groups compared.

A 95% Confidence Intervals (CIs) were computed to accompany the point estimates of stunting burden. As mentioned above, the CIs for D and PAR should not include 0 to conclude that there is inequality. On the other hand, the CIs for PAF and R should not contain 1 to declare the presence of stunting inequality between groups compared. To appreciate the overtime alteration in stunting inequality, the CIs of two consecutive survey years should not overlap.

### Ethical consideration

The analyses were completed using the publicly available data from demographic health surveys. Ethical procedures were the responsibility of the institutions that commissioned, funded, or managed the surveys. All MICS surveys are approved by ICF international as well as an Institutional Review Board (IRB) of the country to ensure that the protocols are in compliance with the U.S. Department of Health and Human Services regulations for the protection of human subjects.

## Results

Table 1 presents the prevalence of stunting disaggregated by the five dimensions of inequality for each of the survey years.

**Table 1:** Over time trends of the childhood stunting disaggregated by the different sub-populations in Mauritania, 2007–2015.

Dimensions	Subgroups	2007		2011		2015	
		Estimate (95% CI)	Sampled Population	Estimate (95% CI)	Sampled Population	Estimate (95% CI)	Sampled Population
**Economic status**	Qui.1 (poorest)	37.77(35.36,40.24)	1977	38.94(36.07, 41.90)	1906	36.60(34.07, 39.19)	2353
	Quintile 2	38.16(35.43, 40.95)	1613	34.47(32.04, 36.99)	1790	31.12(28.56, 33.80)	2204
	Quintile 3	32.42(29.61, 35.35)	1478	29.29(26.88, 31.83)	1619	27.43(25.16, 29.82)	1962
	Quintile 4	24.23(21.84, 26.78)	1500	24.26(21.80, 26.90)	1577	23.70(20.96, 26.67)	1893
	Qui. 5 (richest)	21.06(18.76, 23.55)	1407	17.72(15.34, 20.38)	1387	18.73(16.08, 21.71)	1687
**Educational level**	No formal education	34.24(31.87, 36.70)	2454	32.90(30.56, 35.34)	2281	29.02(26.84, 31.29)	2950
	Primary	29.79(27.38, 32.33)	2312	28.84(26.87, 30.89)	2641	33.37(30.67, 36.18)	2367
	Secondary +	19.94(16.95, 23.31)	970	20.69(18.09, 23.56)	1265	28.24(26.23, 30.34)	3299
**Residence**	Rural	35.72(33.91, 37.58)	4701	32.69(30.97, 34.47)	5025	31.94(30.23, 33.69)	5750
	Urban	25.10(23.07, 27.23)	3276	25.18(23.28, 27.17)	3257	23.30(21.32, 25.41)	4351
**Sex**	Female	29.69(27.87, 31.57)	3916	28.26(26.67, 29.91)	4112	26.48(24.83, 28.20)	5067
	Male	32.97(31.26, 34.73)	4061	31.19(29.52, 32.92)	4169	29.97(28.22, 31.77)	5033
**Subnational region**	Hodhecharghi	38.98(35.07, 43.03)	973	42.95(38.24, 47.79)	893	42.76(38.56, 47.07)	1301
	Hodh elgharbi	34.27(30.48, 38.28)	759	38.68(33.56, 44.06)	798	30.82(27.02, 34.89)	1078
	Assaba	35.96(32.01, 40.11)	886	33.96(29.93, 38.24)	1022	30.10(26.95, 33.45)	1210
	Gorgol	34.95(29.51, 40.81)	795	29.95(25.39, 34.93)	621	23.02(20.60, 25.63)	1243
	Brakna	31.34(27.54, 35.42)	725	22.90(19.11, 27.20)	781	27.99(23.97, 32.39)	932
	Trarza	26.12(22.77, 29.79)	806	21.99(18.57, 25.83)	756	27.06(22.72, 31.89)	682
	Adrar	40.11(33.59, 46.99)	142	33.12(27.22, 39.59)	177	26.40(23.06, 30.03)	39
	nouadhibou	21.72(18.77, 24.99)	270	18.60(14.68, 23.28)	246	14.49(11.29, 18.42)	293
	Tagant	40.62(32.55, 49.23)	160	34.21(28.75, 40.12)	198	37.74(31.96, 43.89)	46
	Guidimagha	40.19(35.78, 44.75)	481	32.70(29.04, 36.58)	737	29.57(24.81, 34.82)	851
	Tiris zemmour	41.48(35.55, 47.66)	114	27.41(21.73, 33.93)	145	21.24(15.11, 29.00)	40
	Inchiri	27.03(19.88, 35.60)	17	23.03(20.61, 25.63)	1903	16.45(9.97, 25.92)	11
	Nouakchott	21.72(19.24, 24.42)	1845	NA	NA	22.47(19.56, 25.69)	2370
**Prevalence**		31.3%,		29.7%		28.2%	

NA: Not applicable, CI: Confidence interval

The population share for each subcategory of the five inequality dimensions has also been presented together with the national stunting prevalence. Across the three waves of the surveys, a total of 26,358 populations were participated. Of them, 13,095 (49.6%), 15,476 (58.7%), 7,685 (29.1%) and 6,236 (23.6%) were females, rural residents, non-educated and in the poorest categories, respectively.

At the national level, there were 31.3%, 29.7% and 28.2% stunted children in 2007, 2011 and 2015, respectively, indicating that no significant reduction was observed over time. The prevalence of stunting among some of the subpopulation was larger than that of the national average. Our disaggregation analysis showed that stunting was more concentrated among the most disadvantaged subpopulations; children born to women in the poorest and non-educated categories, to women who live in rural settings and certain geographical areas. Our study also revealed that male children endured higher burden of stunting than their female counterparts (Table 1).

**Fig 1:**
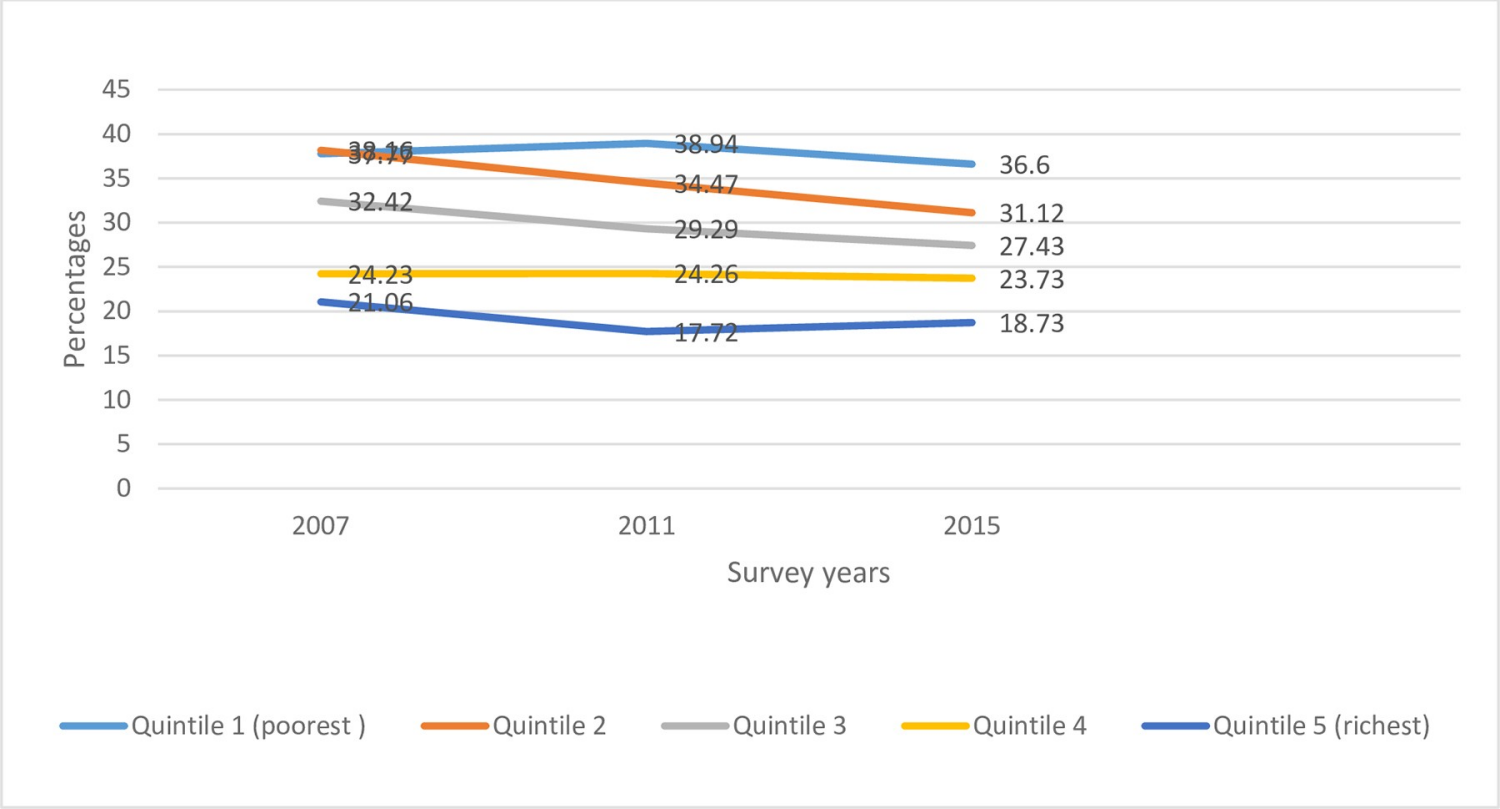
Trends in the prevalence of childhood stunting across the wealth quintiles in Mauritania, MICS (2007–2015).

In terms of change in stunting burden with time, some population groups performed better than others. The prevalence of stunting among children in the quintiles 1, 4 and 5 did not improve with time. However, the other two subgroups of the wealth index saw significant fall during the same period (Fig 1 and Table 1).

**Fig 2:**
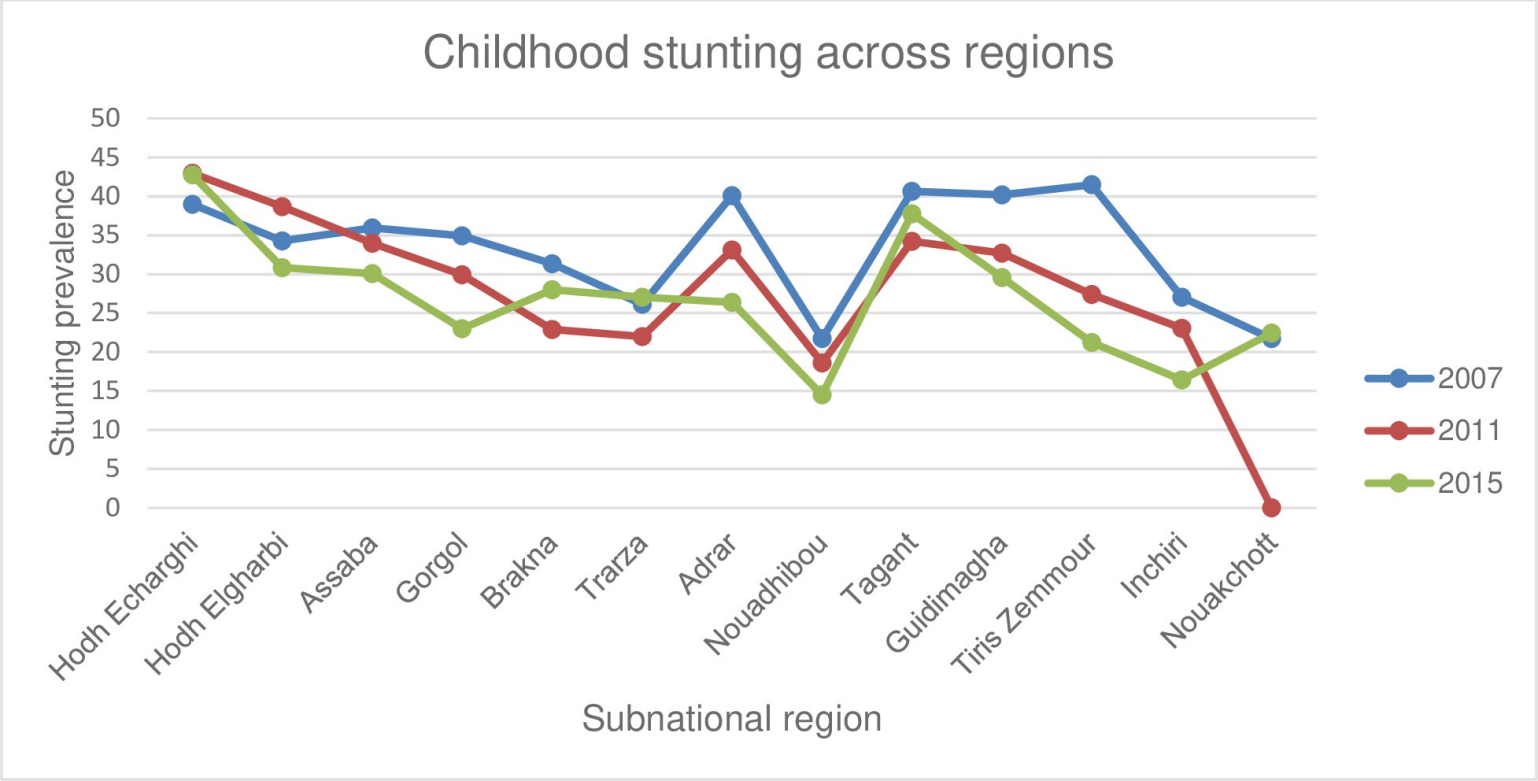
Trends in the prevalence of childhood stunting across the subnational regions in Mauritania, MICS (2007–2015).

Regarding the sub-national regional distribution of the stunting prevalence, we showed that some regions saw a larger fall during the study periods while in others, little increment was observed during the same time period (Fig 2).

Stunting among male and female children had decreased by about three percentage points between the first and the last survey. The fact that the pace of fall of stunting in both sexes is essentially the same could explain for male-female stunting inequality to persist throughout the study period (Table 1). We observed from the findings that the differential performance of the different subgroups over time resulted in stunting to be more concentrated among certain groups than in others (Table 2).

**Table 2:** Extent and over time trends of the socioeconomic and geographic inequalities in the childhood stunting in Mauritania, 2007–2015.

Dimension		2007	2011	2015
	Measures	% (95%CI)	% (95%CI)	% (95%CI)
**Household wealth index**	D	16.70 (13.29, 20.12)	21.22 (17.38, 25.07)	17.86 (14.07, 21.65)
	PAF	-32.84 (-39.17, -26.51)	-40.41 (-46.78, -34.04)	-33.60 (-39.79, -27.42)
	PAR	-10.30 (-12.28, -8.31)	-12.02 (-13.91, -10.12)	-9.48 (-11.23, -7.73)
	R	1.79 (1.55, 2.02)	2.19 (1.84, 2.55)	1.95 (1.63, 2.27)
**Maternal educational level**	D	14.30 (10.31, 18.28)	12.21 (8.58, 15.83)	0.77 (-2.23, 3.79)
	PAF	-33.59 (-41.43, -25.75)	-27.82 (-34.96, -20.68)	-5.60 (-9.68, -1.52)
	PAR	-10.08 (-12.44, -7.73)	-7.97 (-10.02, -5.93)	-1.67 (-2.89, -0.45)
	R	1.71(1.41, 2.01)	1.58 (1.35, 1.82)	1.02 (0.91, 1.13)
**Place of residence**	D	10.62 (7.85, 13.39)	7.51(4.90, 10.13)	8.63 (5.96, 11.29)
	PAF	-19.96 (-23.76, -16.16)	-15.33 (-19.36, -11.30)	-17.40 (-20.91, -13.89)
	PAR	-6.26 (-7.45, -5.06)	-4.56 (-5.75, -3.36)	-4.91 (-5.90, -3.92)
	R	1.42 (1.28, 1.56)	1.29 (1.17, 1.42)	1.37 (1.22, 1.51)
**Child sex**	D	3.28 (0.75, 5.81)	2.92 (0.58, 5.27)	3.48 (1.04, 5.91)
	PAF	-5.33 (-8.63, -2.02)	-4.95(-8.29, -1.62)	-6.15 (-9.24, -3.05)
	PAR	-1.67 (-2.70, -0.63)	-1.47 (-2.46, -0.48)	-1.73 (-2.61, -0.86)
	R	1.11(1.02, 1.20)	1.10 (1.01, 1.19)	1.13 (1.03, 1.22)
**Sub-national region**	D	19.76 (13.16, 26.35)	24.35 (17.93, 30.76)	28.27 (22.73, 33.80)
	PAF	-30.73 (-36.17, -25.30)	-37.44 (-53.78, -21.10)	-48.63 (-62.96, -34.30)
	PAR	-9.64 (-11.34, -7.93)	-11.13 (-15.99, -6.27)	-13.72 (-17.77, -9.68)
	R	1.90 (1.54, 2.26)	2.30 (1.71, 2.89)	2.95 (2.17, 3.72)

**Difference (D)** is a simple, unweighted measure of inequality that shows the absolute inequality between two subgroups. **Ratio (R)** is a simple, unweighted measure of inequality that shows the relative inequality between two subgroups. The **population attributable fraction (PAF)** and **population attributable risk (PAR)** are a complex, weighted measures of inequality that shows the potential for improvement in the national level of a health indicator (stunting), (in relative terms for PAF), that could be achieved if all subgroups had the same level of health (stunting) as a reference subgroup. CI: Confidence interval.

### Magnitude and time trends of stunting inequalities

Stunting inequalities by the different summary measures was presented in Table 2. The study found substantial absolute (D, PAR) and relative (R, PAF) wealth related inequality in stunting in all the three rounds of the MMICS. The large overlap in the CIs of the three MDHS indicates that the poor-rich disparity did not improve over time. Similarly, there is both absolute and relative educational status inequality in stunting in Mauritania though the simple measures failed not show any disparity in 2015. The educational inequality was constant from 2007 to 2011 and considerable reduction was observed between 2011 and 2015. For the simple measures, this means that education related stunting inequality changed from high inequality in 2011 to no inequality in 2015.

The study also indicated large absolute and relative urban-rural gap in stunting in all the rounds. The residence related inequality was generally constant over time by R, D and PAF measures. However, with PAR measure, it fell from 2007 to 2011 and then remained constant until 2015. The sex differentials of stunting had been established in our study, with males experiencing consistently higher prevalence of stunting throughout the study period. Compared with the socioeconomic and urban-rural inequalities, however, sex inequality was not much pronounced. Concerning changes over time, sex inequality did not change with time. We also showed stark absolute and relative regional inequality in stunting in all the study time points, with the gap overall worsen with time.

## Discussion

Following equity analysis techniques recommended by the WHO, we conducted in-depth assessment of the stunting inequality in Mauritania. Overall, we showed stark inequalities in stunting prevalence and the inequality had not seen sign of improvement with time. Children born to women who are economically worse-off, uneducated and live-in rural areas and some regions experienced the disproportionate share of stunting. In all the surveyed period, male children endure the higher share of stunting without the disparity narrowed over the course of time.

Based on difference as a measure of absolute health inequality, the economic status-based inequality indicated that childhood stunting is more pronounced among children in the economically worse-off household in each of the three Mauritania MICS. The pattern of economic status inequality using difference as measure of inequality was constant overtime. Likewise, the other three measures namely PAF, PAR and R also proved the existence of economic related inequalities in all the three rounds with constant pattern overtime. For instance, the value -33.6% of PAR in the 2015 survey indicated that a significant proportion of childhood stunting was concentrated among the subpopulation categorized towards poorer end of the wealth index. This means that the national childhood stunting prevalence would have been decreased by 33.6 percentage points, based on the point estimate, had the prevalence of stunting among the four subcategories of wealth was similar to that of the richest wealth quintile. Concordance with our findings, prior study has shown that stunting disproportionately impacts poor children [18]. The poor-rich inequality might be due to inequalities in economic status, educational status, institutional delivery, maternal age at birth, household sanitation, and due to geographical disparities [19].

In agreement with prior evidence [20, 21], the study showed the existence of education-based inequality in the prevalence of stunting in all the studied years using the complex measures, with children born to non-educated mothers being at higher risk. As maternal educational status increases beyond primary schooling, undernutrition tends to decrease [22]. This finding suggests that increasing the coverage of secondary or more education is important to sufficiently reduce the within country stunting burden. The educational inequality in the burden of stunting could partly be explained by the fact that, positive relationship exists between maternal educational and household wealth [23]. Interestingly, among more educated mothers, household wealth tends to prevent the occurrence of stunting and other nutritional problems.

The present study found a higher prevalence of stunting in rural area by all the measures of inequalities and this urban-rural difference persisted over time. For instance, the inequality measure of Difference in the most recent survey indicated that childhood stunting among the rural residents was higher by nearly 9 percentage point, on average, as compared to the urban residents. The pro-urban scenario of stunting in our study was similarly recorded in a prior study [24]. This urban-rural difference could be a result of differences in the socioeconomic conditions and prenatal care between urban and rural settings [25]. In contrast to our findings, however, Chirande L et al showed that children in urban areas had higher odd of stunting than children living in rural settings [26], indicating that urban children are not always better than their rural counterparts in terms of chronic malnutrition.

Similarly, we recorded substantial sub-national regional inequality in all the rounds using all the measures. For instance, the Difference measure indicated that more than 28 percentage point difference was observed between the region with the highest childhood stunting prevalence (Hodhecharghi) and the region with the lowest prevalence (Novadhibou) in the most recent survey. Furthermore, the PAF measure proved the presence of significant inequalities across the sub-national regions within the country. According to this measure, the national prevalence of stunting in 2015 could have been fallen by, based on the point estimate, 48.6% had stunting level among the other regions been reduced to a level in Novadhibou region. Not only does inequality exist across the regions in all the waves of the MICS in Mauritania, the inequality seemed to have increased when we compared the first and the last rounds. The unequal rate of reduction of stunting in different regions in different time period could underlie for the inequality to persist and continued to grow over time. For instance, even if the prevalence was nearly similar in the first rounds of the MICS (2007), prevalence of childhood stunting in Tris Zemmour region was decreased by more than 20 percentage point from 2007 to 2015 survey. However, it was increased by just nearly 4 percentage point in Hodh Echarghi region. Our finding is in agreement with that of a previous study done in Democratic Republic of Congo, where childhood stunting was varied by the sub-national regions within in the country [27]. The observed regional variation could be liked with differences in nutrition intake related culture, availability of food, and agricultural activities and [27]. Moreover, it has been shown that socioeconomic inequality of stunting was shown to vary by the subnational regions [28], showing the differential impact of sub-national regions on wealth and education related inequalities in stunting.

Finally, we showed pro-female condition in the burden of stunting, where male children had higher chance of being stunted then female children. Existing body of evidence in Sub-Saharan Africa supports our conclusion on this nature of the sex differential of stunting [29]. Further studies are required on the underlying reasons for the observed sex differential of stunting. Available literature suggests that sex differential of timing of complementary feeding may drive sex inequality in stunting; boys were found to have started complementary feeding at 2–3 month than girls do, and to eat meals with complementary feeding [30]. Another evidence suggests that initiating complementary feeding early was associated with lower height-for-age compared to the fully breast-fed children [31], suggesting that starting additional foods in addition to breast milk early is likely to cause stunting to be more common among boys.

The study has some strengths. Our study has improved the existing knowledge in many ways. First, we examined the time trend of stunting inequality. Since prior efforts are restricted to one-time cross-sectional analysis of stunting, studies that show how stunting inequality evolves over time is important to reframe future implementations of equity interventions. Second, we strictly followed the standard procedures in the analysis of health care inequalities as stipulated in the WHO health inequality analysis book [5]. Further, in compliance with the WHO recommendation, we calculated simple, complex, relative and absolute summary measures. The purpose of adopting different inequality measures in single study is that, it would help researchers to interpret findings from various perspectives and dimensions. Also, the method of using both relative and absolute measures give researchers the opportunity not to miss out inequality in a health care indicator; inequality may exist in absolute measures but not in relative measures and vice versa. Finally, we are confident that the use of the high-quality WHO Health Equity Monitor (HEM) dataset for our study has undoubtedly increased quality of the findings.

The limitations of the study include, the findings could not be generalized to settings other than the urban and rural settings as well as the sub-national regions. Moreover, the WHO equity monitor database does not provide information on stunting burden by different age groups. Finally, we did not decompose the observed stunting inequality to the underlying factors that could explain the stunting inequality. We therefore recommend the conduct of a decomposition analysis to better understand why stunting inequality remained in Mauritania between different population groups.

## Conclusions

We had shown huge socioeconomic and area-based childhood stunting inequalities in all the survey years and across all the dimensions of inequality between 2007 and 2015 MMICS. We observed fluctuation in the overtime alteration of stunting inequality; in some equity stratifiers such as sex, the inequality remain unchanged, while in others, there were increasing or decreasing patterns. The subnational regional inequalities increased significantly between the first and the last surveys. Different stakeholders need to work on equitable nutrition interventions that target the subgroups which suffer more from stunting. Further studies are needed to find out the reasons for the stunting inequality using a decomposition technique.

## Supporting information

S1 Appendix Summary measures. (CSV)

S2 Appendix Disaggregated data. (CSV)
